# Resveratrol Can Be Stable in a Medium Containing Fetal Bovine Serum with Pyruvate but Shortens the Lifespan of Human Fibroblastic Hs68 Cells

**DOI:** 10.1155/2018/2371734

**Published:** 2018-05-10

**Authors:** Yuan-Jhe Chang, Ya-Chun Chang, Rosa Huang Liu, Chia-Wen Chen, Inn Lee, Nae-Cherng Yang

**Affiliations:** ^1^Institute of Medicine, Chung Shan Medical University, Taichung, Taiwan; ^2^Department of Nutrition, Chung Shan Medical University, Taichung, Taiwan; ^3^Department of Nutrition, Chung Shan Medical University Hospital, Taichung, Taiwan

## Abstract

This study is aimed at developing a method that can inhibit resveratrol (Res) degradation in Dulbecco's modified Eagle medium (DMEM) and at evaluating the effects of Res on the replicative lifespan of Hs68 cells. We hypothesized that Res can extend the lifespan of Hs68 cells if we can inhibit the oxidative degradation of Res in the medium. We found that the addition of ≥5 U/mL SOD to the medium could completely inhibit Res degradation in DMEM. Fetal bovine serum (FBS) contained 29.3 ± 1.1 U/mL of SOD activity. FBS could prevent Res degradation in the medium through SOD activity and Res–FBS interaction, but the regular FBS concentration (i.e., 10% FBS) exhibited an insufficient ability to completely inhibit Res degradation. We found that pyruvate (1 mM) could potentiate SOD to scavenge superoxide at approximately 2.2-fold. Thus, 10% FBS combined with pyruvate (1 mM) could completely inhibit Res degradation. When Res was not degraded, it still shortened the lifespan of Hs68 cells. Overall, the proposed method involving 10% FBS combined with pyruvate (1 mM) could completely prevent Res degradation. However, in contrast to our hypothesis, Res could induce the shortening of the lifespan of Hs68 cells. The stability of Res analogs (i.e., oxy-Res and acetyl-Res) in the medium and their effects on the lifespan of Hs68 cells were also investigated.

## 1. Introduction

Resveratrol (Res) is a polyphenol that exists in grape skins and is present in red wine. This micronutrient possesses antioxidant, anti-inflammatory, antidiabetic, anticancer, and cardiovascular protective properties [1, 2]. A calorie restriction mimetic (CRM) is a compound that mimics the metabolic, hormonal, and physiological effects of calorie restriction (CR), activates stress response pathways observed in CR, enhances stress protection, produces CR-like effects on longevity, reduces age-related disease, and maintains youthful function [3]. All of these actions occur without significantly reducing food intake, at least initially [3]. Whether Res is a CRM is still unclear [3]. Several studies demonstrated that Res can activate sirtuins (e.g., human SIRT1) through an indirect mechanism [4, 5], which suggests its CRM potential [6–8]. In addition, the current literature is inconsistent regarding whether Res can extend the lifespan or retard the senescence of human cells [9–16], suggesting that the difference in cell types used may affect the effects of Res on cellular senescence. We recently demonstrated that glucose restriction can mimic CR to extend the lifespan of human fibroblastic Hs68 cells [17]. Theoretically, if Res possesses CRM potential, then it should extend the lifespan of Hs68 cells. Hence, we aimed to clarify the CRM potential of Res by examining whether Res can extend the lifespan of Hs68 cells.

Additionally, polyphenols are oxidized in medium, and they generate H_2_O_2_, which interferes with experiments and yields artificial results by inducing oxidative stress in cell model studies [18, 19]. Therefore, the nonoxidation of Res in medium must be confirmed when using a cell model to evaluate the CRM potential of Res. Our previous study revealed that Res is degraded by oxidation in Basal Medium Eagle (BME) [19]. We found that 96% of Res is degraded, and approximately 90 *μ*M H_2_O_2_ is generated when 200 *μ*M Res is incubated in BME at 37°C for 24 h [19]. Nonetheless, the extent of Res degradation in Dulbecco's modified Eagle medium (DMEM), which is a culture medium for Hs68 cells, is unclear. If Res is degraded in DMEM, then a method to prevent the oxidative degradation of Res in this medium should be developed. Hence, Sang et al. [20] reported that 20 *μ*M of EGCG is almost not degraded when it is incubated in HAM's F12 : RPMI 1640 (1 : 1) medium with 5 U/mL superoxide dismutase (SOD) for 6 h. Currently, we aim to determine whether SOD can inhibit the degradation of Res in DMEM. Moreover, fetal bovine serum (FBS), a commonly used serum, increases the stability of Res in a solution via a polyphenol–protein interaction [21], suggesting that FBS may inhibit Res degradation by forming a Res–FBS complex in media. In addition, human and animal sera exhibit SOD activity. We also aim to verify whether the SOD activity in FBS in a cell culture is sufficient to inhibit Res degradation in DMEM. The addition of catalase or pyruvate to this medium to scavenge the produced H_2_O_2_ should help eliminate artificial effects caused by Res oxidation in the culture medium. Nevertheless, the ability of SOD or FBS to inhibit Res degradation in the presence of catalase or pyruvate in media is still unclear.

Given the low solubility, low bioavailability, and high metabolism of Res [22], recent interest in Res research is being extended to its naturally occurring derivatives, namely, oxyresveratrol (oxy-Res) [23] and acetyl-resveratrol (ac-Res) [24]. Oxy-Res is a naturally occurring Res analog present in mulberry fruit (*Morus alba* L.). With a hydroxyl group added to the aromatic ring of Res, this analog possesses improved water solubility [23] and antioxidant activity [25] compared with Res. Ac-Res is another naturally occurring Res analog found in marine invertebrates [24]. Res acetylation increases the lipophilic properties, its cellular uptake in vitro [26], and its bioavailability in vivo [27]. However, the degradation inhibition of oxy-Res and ac-Res in DMEM and their effects on the lifespan of Hs68 cells are unknown.

In the present study, we aimed to develop a method to inhibit Res degradation in DMEM under regular culture conditions and reveal the effects of Res on the lifespan of human Hs68 cells. We hypothesized that Res will extend the lifespan of Hs68 cells if the oxidative degradation of Res in the medium can be avoided. Oxy-Res and ac-Res degradation in the medium and their effects on the Hs68 cell lifespan were also investigated. The physiological level of Res in human blood can reach approximately 3 *μ*M [1, 28]. Thus, we tested Res and its analogs at a concentration range of 0–20 *μ*M. We used the cumulative growth curve to monitor cell lifespan; senescence-associated *β*-galactosidase (SA-*β*G) activity was used as the biomarker of cell senescence [29, 30]. The degradation of Res, oxy-Res, and ac-Res was detected by high-performance liquid chromatography (HPLC). We used a cell-free system to evaluate the degradation of Res and its analogs in medium because the remaining level of Res will be affected with the cellular uptake of Res and its analogs. In addition, CR extends the lifespan of human lung fibroblastic IMR-90 cells [31]. Thus, we hypothesized that Res and its analogs can extend the replicative lifespan of IMR-90 cells.

## 2. Materials and Methods

### 2.1. Chemicals and Materials

All chemicals used were of analytical grade. NaCl, KCl, NaOH, MgCl∙6H_2_O, Na_2_HPO_4_, KH_2_PO_4_, NaHCO_3_, and dimethylsulfoxide (DMSO) were purchased from Merck (Darmstadt, Germany). *N*,*N*-Dimethyl formamide, citric acid, potassium ferricyanide, potassium ferrocyanide, glutaraldehyde, 3-(4,5-dimethylthiazol-2-yl)-2,5-diphenyltetrazolium, 5-bromo-4-chloro-3-indolyl *β*-D-galactopyranoside (X-Gal), SOD (Cat. no. S7571), catalase (Cat. no. C1345), sodium pyruvate, and other chemicals were obtained from Sigma Chemical Corp. (St. Louis, MO, USA). Fluorescein di-*β*-D-galactopyranoside (FDG) was purchased from Molecular Probes (Eugene, OR, USA). High-purity Res (≥99%), oxy-Res (≥98%), and ac-Res (≥98%) were obtained from Great Forest Biomedical Ltd. (Shanghai, China). DMEM (Cat. no. SH30003.02), Eagle's minimum essential medium (MEM) (Cat. no. SH30008.02), trypsin, penicillin–streptomycin, and nonessential amino acids were purchased from HyClone (Logan, UT, USA).

### 2.2. Determination of Res, Oxy-Res, and Ac-Res Degradation by HPLC

Res and its analogs were analyzed by a reverse-phase HPLC system consisting of a Shimadzu system controller (Osaka, Japan), Shimadzu LC-10AD pump, Sharpsil-UC18 column (4.6 mm × 250 mm, 5 *μ*m), and Shimadzu SPD-10A UV-VIS detector. The elution program was set as 100% solvent A (0.2% trifluoroacetic acid) for 5 min and progressed to 100% solvent B (methanol) from 5 min to 20 min at a flow rate of 1 mL/min. Absorbance signals were detected at 306 nm. The sample loop volume was 20 *μ*L.

### 2.3. SOD Activity Assay

SOD activity was detected using a nonenzymatic colorimetric method with some modifications [32]. The total volume of 200 *μ*L reaction buffer contained 20 *μ*L of NBT (0.5 mM), 20 *μ*L of NADH solution (1.54 mM), 33.3 *μ*L of Tris-HCl buffer (16 mM, pH 8.0), 40 *μ*L of FBS, and 53.7 *μ*L of pure water. To obtain the calibration curve, FBS and pure water volumes were used, instead of 93.7 mL of SOD standard solution with 0–12.5 U/mL SOD activities. The reaction was initiated by adding 33.3 *μ*L of phenazine methosulfate solution (0.12 mM) into the well. After incubation at 25°C for 5 min, absorbance at 570 nm was measured against a blank sample. The inhibition percentage [33] was calculated using the following equation:[1–(sample OD_570_–blank OD_570_/zero–SOD OD_570_–blank OD_570_)] × 100%. A four-parameter logistic curve fit algorithm was used for the quantitative analysis of SOD activity.

### 2.4. Cell Culture

Human fibroblastic Hs68 cells (ATCC# CRL-1635) and IMR-90 cells (ATCC# CCL-186) were purchased from the Cell Culture Center of the Food Industry Research and Development Institute (Hsinchu, Taiwan). Hs68 is one of the series of human foreskin fibroblast lines developed at the Naval Biosciences Laboratory in Oakland, California. Hs68 was obtained from an apparently normal Caucasian newborn male with a finite lifespan. Hs68 cells were regularly cultured in DMEM in 75 cm^2^ flasks with 10% FBS, 3.7 g/L sodium bicarbonate, and antibiotics at 37°C in a humidified incubator with 5% CO_2_. IMR-90 cells were obtained from the lungs of a 16-week-old female human fetus. IMR-90 cells were regularly cultured in MEM with 10% FBS, 2.2 g/L sodium bicarbonate, 0.1 mM nonessential amino acid, and antibiotics.

### 2.5. Cytotoxicity Assays

Cytotoxicity was assayed using the lactate dehydrogenase (LDH) Cytotoxicity Assay Kit (Cayman Chemical, Ann Arbor, MI) with some modifications. Briefly, the cells were cultured in 12-well plates until confluence and incubated with the tested agents for 24 h. LDH-containing medium (200 *μ*L) was transferred into a 96-well microplate and mixed with the LDH reaction solution. After incubation for 30 min at room temperature, the absorbance was measured at 490 nm using a microplate reader. LDH release (%) is expressed as a percentage of the control.

### 2.6. Cumulative Growth Curve Assay

Cells were serially cultured in a 10 cm dish with 1.0 × 10^5^ cells per dish in 10 mL of cultured medium. The cells were subcultured once a week, and the cell numbers were counted by trypan blue exclusion assay. The population doubling levels (PDLs) were calculated as log_2_ (Nt/No) [17], where Nt and No are the total cell counts at harvesting and seeding, respectively. The cumulative PDLs (CPDs) were obtained by summation of total PDL before a given passage time. The replicative lifespan was determined when cells were senescent and defined as a Nt/No ratio of less than 1 at a certain passage or less than 1.5 for two consecutive passages. The precise CPDs of Hs68 and IMR-90 cells were not specified by the suppliers. Therefore, we defined CPDs at the initial passage as zero. We used the additional CPDs to represent the doubling levels after the initial passage.

### 2.7. Determination of SA-*β*G Activity

SA-*β*G activity was measured using a double-substrate assay [29]. The monolayer of cells (5 × 10^4^) cultured in a 12-well plate overnight was washed two times in phosphate-buffered saline (PBS; pH 7.4) and subsequently fixed for 5 min in 2.0% formaldehyde and 0.2% glutaraldehyde buffered with PBS. After being washed three times in PBS, the fixed cells were incubated in the staining solution containing X-Gal (2.45 mM, pH 6.0, freshly prepared) and FDG (40 *μ*M) in a humidified incubator at 37°C for 24 h without CO_2_. After incubation, 100 *μ*L of the supernatant was transferred into a 96-well plate for fluorescent measurement in triplicate. The fluorescein fluorescence was measured using a fluorometer (Flx800, Bio-Tek Instruments Inc., Winooski, VT, USA) with excitation and emission at 485 and 535 nm, respectively. The X-Gal-stained cells were further photographed under a microscope (Nikon, Japan) at 100x magnification for qualitative detection of SA-*β*G activity. The staining solution was freshly prepared by mixing 3.7 mL of citric acid (0.2 M), 6.3 mL of Na_2_HPO_4_ (0.4 M), 1 mL of X-Gal (20 mg/mL or 49 mM in *N*,*N*-dimethyl formamide), 1 mL of potassium ferricyanide (100 mM), 1 mL of potassium ferrocyanide (100 mM), 0.6 mL of NaCl (5 M), 0.2 mL of MgCl_2_ (0.2 M), and 6.2 mL of deionized water, which yielded a total volume of 20 mL.

### 2.8. Evaluation of FBS–Res Interaction

FBS in the medium was precipitated with methanol [17]. In brief, 100 *μ*L of 10% FBS in DMEM was mixed with 900 *μ*L of methanol. After centrifugation at 12000 ×*g* was performed for 10 min, the supernatant in a certain volume (*V*) was transferred to a glass tube and dried with N_2_ gas. Afterward, *V*/10 volumes of pure water were added to obtain the deproteined sample. The FBS–Res interaction was evaluated on the basis of the remaining Res level (%) of the deproteined sample through HPLC.

### 2.9. Data Analysis

Data were analyzed using analysis of variance (ANOVA) followed by the least significant difference test for group mean comparison. Student's *t*-tests were used to identify statistically significant differences between two groups. All analyses relied on SPSS v 17.0 software (SPSS Inc., Chicago, IL, USA). *P* values less than 0.05 were considered statistically significant.

## 3. Results

### 3.1. Chromatograms of Res, Oxy-Res, and Ac-Res Standards

**Figure 1:**
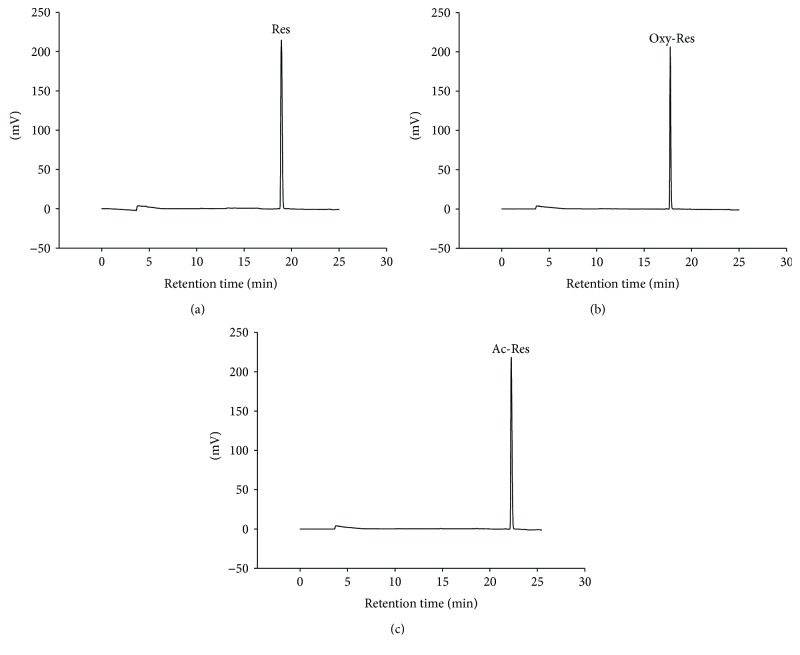
Chromatograms of (a) resveratrol (Res), (b) oxyresveratrol (oxy-Res), and (c) acetyl-resveratrol (ac-Res) standards. Each standard (200 *μ*M) was analyzed in dimethylsulfoxide (DMSO) with our high-performance liquid chromatography (HPLC) method.

Figures 1(a)–1(c) show the chromatograms of Res, oxy-Res, and ac-Res standards detected by our HPLC method, respectively. The retention times for oxy-Res, Res, and ac-Res were 17, 18, and 22 min, respectively.

### 3.2. Effects of SOD on Res Degradation in DMEM

As shown in Table 1 (upper panel), Res was unstable in DMEM in the culture oven at 37°C supplied with 5% CO_2_. Approximately 62.1% of Res was degraded after the seven-day incubation. SOD can effectively inhibit the degradation of Res in DMEM. Addition of ≥5 U/mL SOD into the medium can completely inhibit Res degradation.

### 3.3. Effects of FBS on Res Degradation in DMEM

FBS also remarkably inhibited Res degradation (Table 1, lower panel). Only 20.6% of Res was degraded after the 7-day incubation. This result suggested that the SOD activity of 10% FBS and the Res–FBS interaction mechanism in DMEM were insufficient to inhibit Res degradation completely. We also evaluated the additional requirement of SOD for the complete inhibition of Res degradation in a medium already containing 10% FBS. The results showed that ≥5 U/mL SOD to the medium should still be added to inhibit Res degradation completely (Table 1).

### 3.4. Effects of Catalase and Pyruvate on Res Degradation in DMEM

We aimed to develop a method to inhibit Res degradation using a medium containing catalase or pyruvate, which can help eliminate the possible artificial effects caused by the H_2_O_2_ generated from the Res oxidation. However, we found that catalase and pyruvate potentiated Res degradation in DMEM (Table 2).

### 3.5. Additional SOD Required to Inhibit Res Degradation in DMEM Containing FBS and Catalase or Pyruvate

**Figure 2:**
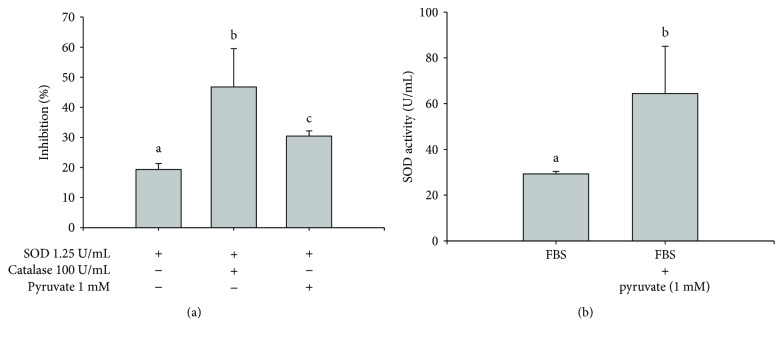
Effect of pyruvate or catalase to potentiate the superoxide dismutase (SOD) activity. (a) SOD (1.25 U/mL) was incubated with or without catalase (100 U/mL) or pyruvate (1 mM) in a microplate in a total volume of 200 *μ*L, as described in Materials and Methods. SOD activities are expressed as percent of inhibition. (b) Effect of pyruvate on the SOD activity of fetal bovine serum (FBS). FBS (40 *μ*L) was incubated with pyruvate (1 mM) in a microplate at a total volume of 200 *μ*L, as described in Materials and Methods. SOD activities are expressed as U/mL. Values (mean ± standard deviation, *n* = 3) without a common letter are significantly different (*P* < 0.05).

We further investigated the additional SOD requirement for complete inhibition of Res degradation in medium containing both 10% FBS and catalase (100 U/mL) or pyruvate (1 mM). Remarkably, we found that no additional SOD was needed to inhibit Res degradation completely. As shown in Table 3, 10% FBS was sufficient to inhibit Res degradation completely in a medium containing catalase (100 U/mL) or pyruvate (1 mM). We further hypothesized that catalase and pyruvate can potentiate the activity of SOD enzyme. Thus, we tested the effects of catalase or pyruvate on the activity of SOD enzyme. We found that SOD activities were significantly increased by catalase and pyruvate (Figure 2(a)). In addition, the SOD activity of FBS was 29.3 ± 1.1 U/mL (Figure 2(b)). Pyruvate at 1 mM can potentiate the SOD activity of FBS (64.4 ± 20.6 U/mL) by about 2.2-fold (Figure 2(b)), thereby demonstrating that pyruvate can also potentiate the SOD activity of FBS. Pyruvate or catalase exhibited no SOD activity (data not shown).

### 3.6. Effects of FBS Combined with Pyruvate on the Degradation of Res in MEM

As shown in Table 4, Res was also unstable in MEM. Res (31%) was degraded after the seven-day incubation. Similarly, we found that 10% FBS combined with pyruvate (1 mM) can completely inhibit the degradation of Res in MEM in the culture oven at 37°C supplied with 5% CO_2_.

### 3.7. Stability of Res, Oxy-Res, and Ac-Res in Medium with FBS and Pyruvate under Regular Culture Conditions

**Figure 3:**
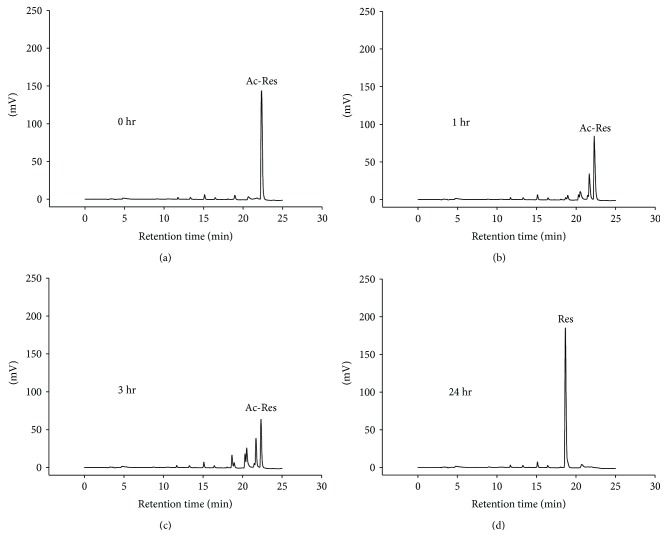
Stability of ac-Res in Dulbecco's modified Eagle medium (DMEM) with FBS and pyruvate. Ac-Res (200 *μ*M) was incubated for 0–24 h in 10 mL of DMEM with 10% FBS and pyruvate (1 mM) under regular culture conditions. At the indicated time point, a proper volume of medium was removed and analyzed by HPLC. The chromatograms show results at (a) 0, (b) 1, (c) 3, and (d) 24 h.

We further confirmed whether Res and oxy-Res were undegraded in DMEM and MEM containing 10% FBS and pyruvate (1 mM) for 1–7 days under regular culture conditions. In addition to containing 10% FBS, a regular culture condition was defined as the medium containing all of the other needed ingredients, including sodium bicarbonate, antibiotics, and nonessential amino acids if necessary, and incubated at 37°C in culture oven supplied with 5% CO_2_. As shown in Table 5, the remaining levels of both Res and oxy-Res after incubation for 1–7 days were similar to those at day 0, with a value of approximately 100% (*P* > 0.05). Res and oxy-Res were also undegraded in MEM with 10% FBS and pyruvate (1 mM) under regular culture conditions (Table 5). These results suggested that the additional ingredients, including antibiotic and nonessential amino acids, exerted no effect on the ability of 10% FBS combined with pyruvate (1 mM) to inhibit Res degradation in DMEM and MEM. Furthermore, we found that the peak of ac-Res retention (at 22 min) decreased with incubation time and changed into a profile with multiple peaks (Figure 3). The newly produced multiple peaks were regarded as Res with different numbers of acetyl groups at different positions. Notably, a single Res peak at 18 min was generated after 24 h of incubation (Figure 3). These results showed that ac-Res was hydrolyzed within 24 h to become Res in DMEM.

### 3.8. Cytotoxic Effects of Res, Oxy-Res, and Ac-Res on Hs68 and IMR-90 Cells

**Figure 4:**
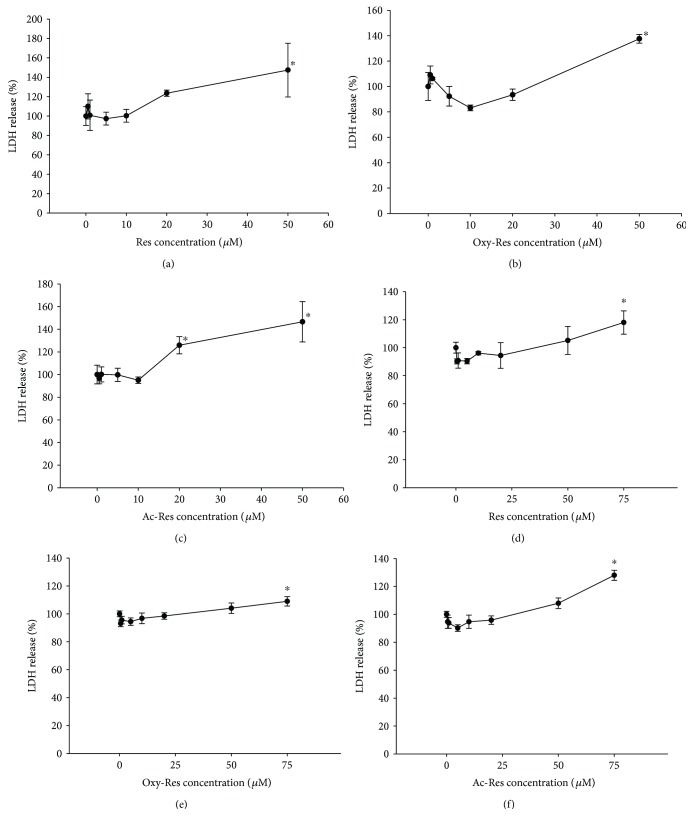
Cytotoxic effects of Res, oxy-Res, and ac-Res on Hs68 and IMR-90 cells. Cells were incubated for 24 h in a medium containing 0–50 *μ*M Res (a), oxy-Res (b), or ac-Res (c) for Hs68 cells and 0–75 *μ*M Res (d), oxy-Res (e), or ac-Res (f) for IMR-90 with 10% FBS and pyruvate (1 mM) under regular culture conditions. Cytotoxicity was evaluated using lactate dehydrogenase-releasing method. Values are expressed as mean ± standard deviation (*n* = 3). Asterisks (^∗^) indicate significant differences (*P* < 0.05) compared with the control group.

We detected the release of LDH from Hs68 and IMR-90 cells to monitor the cytotoxicity of Res and its analogs. In Figures 4(a)–4(c), 50 *μ*M of Res, oxy-Res, and ac-Res induced an increase in LDH release, suggesting that high levels of Res and its analogs induced cytotoxicity in Hs68 cells. Additionally, ac-Res induced a significant release of LDH at a low concentration (20 *μ*M), but this result was not observed at low Res or oxy-Res concentrations. This difference indicated that the cytotoxicity of ac-Res was higher than those of Res and oxy-Res. Furthermore, 5 or 10 *μ*M Res, oxy-Res, and ac-Res showed a slight reduction in the release of LDH, indicating that Res, oxy-Res, and ac-Res exhibited a U-shaped toxicity to Hs68 cells. Res, oxy-Res, and ac-Res were less toxic to IMR-90 cells. As shown in Figures 4(d)–4(f), 75 *μ*M Res, oxy-Res, and ac-Res was needed to induce an increase in LDH release. In addition, 0–75 *μ*M Res, oxy-Res, and ac-Res also showed a U-shaped toxicity to IMR-90 cells.

### 3.9. Effect of Res, Oxy-Res, and Ac-Res on Cell Lifespan

**Figure 5:**
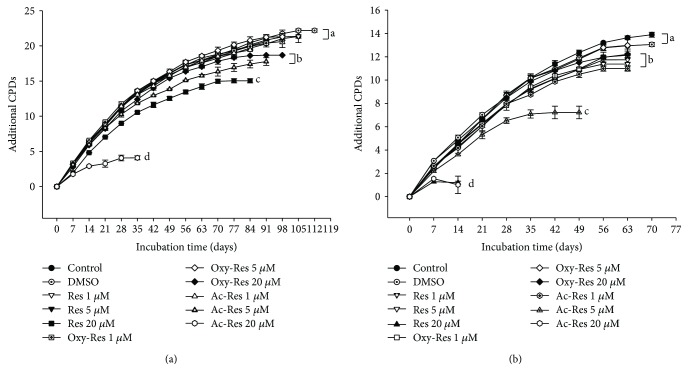
Effects of Res, oxy-Res, and ac-Res on the lifespans of Hs68 and IMR-90 cells. Cells were serially cultured in medium containing 0, 1, 5, and 20 *μ*M of Res, oxy-Res, or ac-Res with 10% FBS and pyruvate (1 mM) under regular culture conditions. The cumulative growth curves were obtained for (a) Hs68 and (b) IMR-90 cells. DMSO represents the solvent control group. Values (mean ± standard deviation, *n* = 3) without a common letter at the same incubation time are significantly different (*P* < 0.05). CPD: cumulative population doubling levels.

We used 0, 1, 5, and 20 *μ*M Res and its analogs to examine their effects on cell lifespan. As shown in Figure 5(a), none of the compounds, namely, Res, oxy-Res, or ac-Res, was able to extend the lifespan of Hs68 cells. Res, oxy-Res, and ac-Res at 0–20 *μ*M caused no increase on CPDs at senescence compared with those of either the control or the solvent control (DMSO). High levels of Res and its analogs shortened the lifespan of Hs68 cells. The cumulative growth curves flattened faster than that of the control. The CPDs at senescence were less than those of the control (*P* < 0.05) and decreased in a concentration-dependent manner. The order of the lifespan-shortening effects was ac-Res > Res > oxy-Res. Similar results were obtained when IMR-90 cells were used to evaluate the effects of Res, oxy-Res, and ac-Res on cell lifespan (Figure 5(b)).

Mikuła-Pietrasik et al. [15] reported that at a concentration of 0.5 *μ*M, Res can retard the senescence of mesothelial cells. Thus, in addition to our original used concentrations, we also assessed the effect of 0.5 *μ*M Res on the senescence of Hs68 cells. We observed that the cumulative growth curve with 0.5 *μ*M Res treatment was similar to those of the control and solvent control (data not shown). Therefore, the low Res concentration (0.5 *μ*M) cannot exert any lifespan-extending effects on Hs68 cells.

### 3.10. Effect of Res, Oxy-Res, and Ac-Res on SA-*β*G Activity

**Figure 6:**
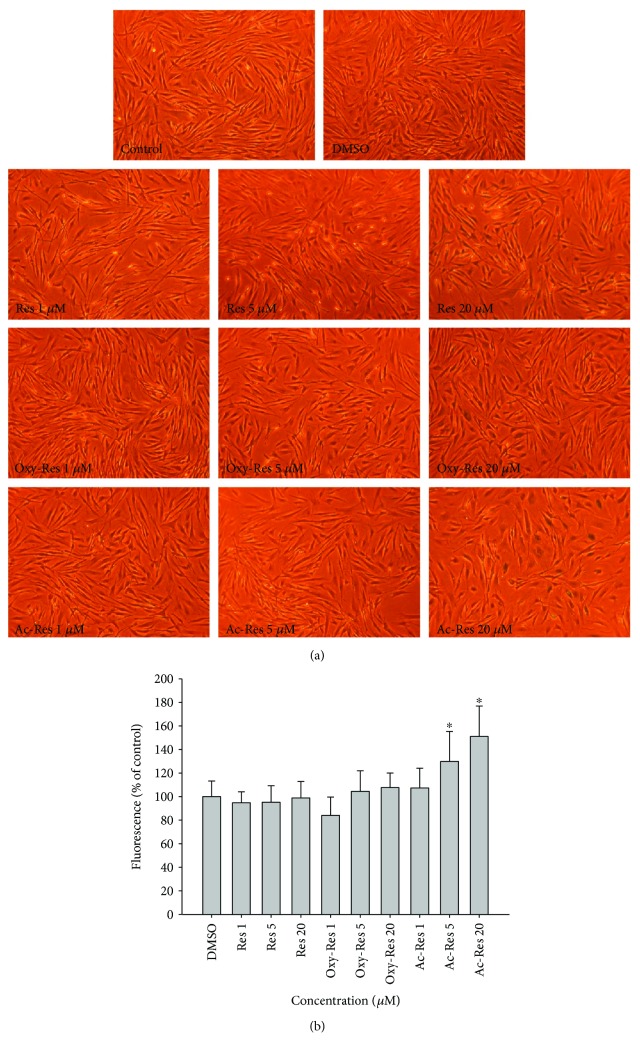
Effects of Res, oxy-Res, and ac-Res on the senescence-associated *β*-galactosidase (SA-*β*G) activity of Hs68 cells. On day 35 of the serially cultured Hs68 cells, SA-*β*G activities were measured using the double-substrate method (i.e., qualitatively measured by 5-bromo-4-chloro-3-indolyl *β*-D-galactopyranoside (X-Gal) staining (a) and quantified by the relative fluorescein fluorescence (b)). Values are expressed as mean ± standard deviation (*n* = 3); asterisks (^∗^) indicate significant differences (*P* < 0.05) compared with the control group.

**Figure 7:**
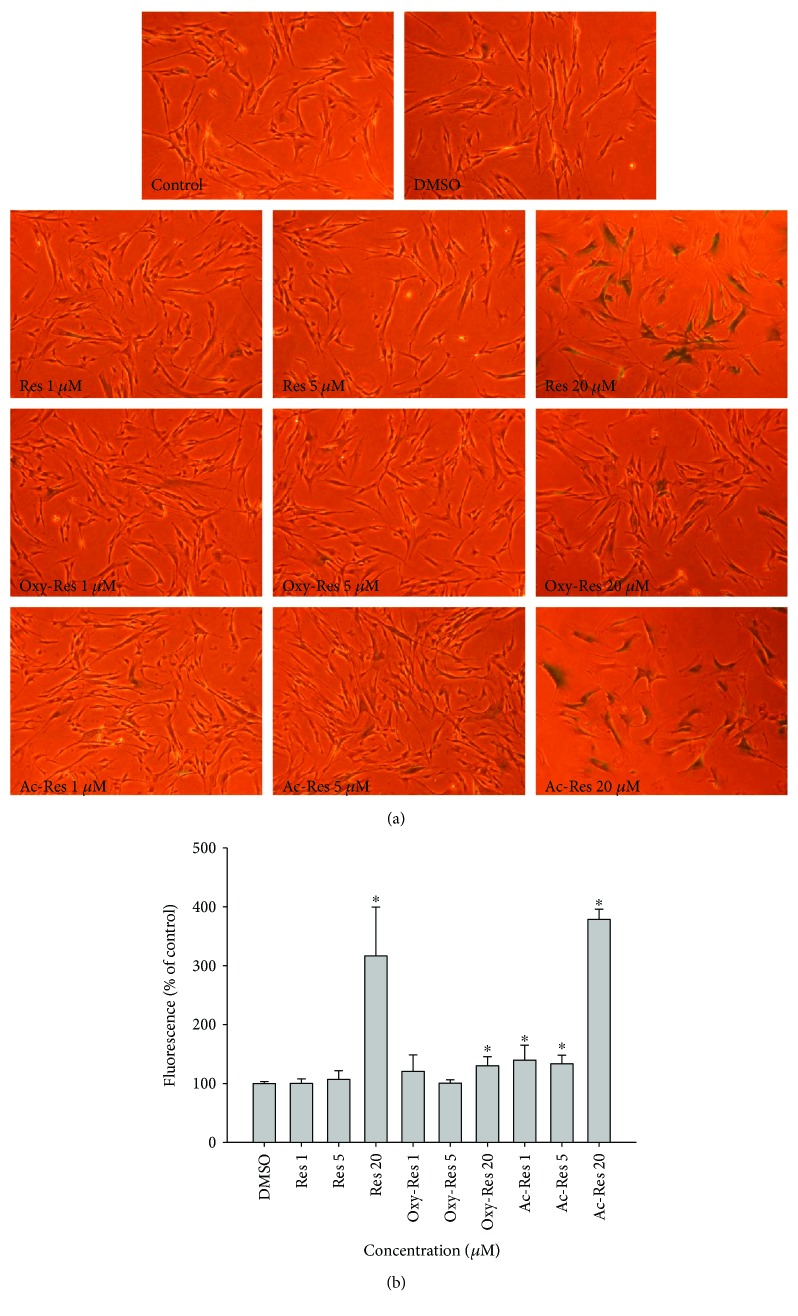
Effects of Res, oxy-Res, and ac-Res on the SA-*β*G activity of IMR-90 cells. On day 14 of the serially cultured IMR-90 cells, SA-*β*G activities were measured by the double-substrate method (i.e., qualitatively measured by X-Gal staining (a) and quantified by the relative fluorescein fluorescence (b)). Values are expressed as mean ± standard deviation (*n* = 3). Asterisks (^∗^) indicate significant differences (*P* < 0.05) compared with the control group.

During the processes of obtaining the cumulative curves at day 35 for the Hs68 cell lifespan assay and day 14 for the IMR-90 cell lifespan assay, we detected the effects of Res, oxy-Res, and ac-Res on SA-*β*G activity in both Hs68 and IMR-90 cells using the double-substrate method, that is, SA-*β*G activity was detected qualitatively by X-Gal staining and quantitatively by an FDG method simultaneously [29]. We found that 20 *μ*M of ac-Res at day 35 induced Hs68 cells with a larger and flatter morphology, stronger blue color (Figure 6(a)), and significantly increased fluorescein fluorescence of the cells (*P* < 0.05) than those of the control (Figure 6(b)). For IMR-90 cells, 20 *μ*M of ac-Res, Res, and oxy-Res can induce a senescent morphology with increased SA-*β*G activity at day 14 during the cumulative growth compared with that of the control (Figures 7(a) and 7(b)). Res and its analogs enhanced the SA-*β*G activity in the following order: ac-Res > Res > oxy-Res.

### 3.11. Uptake of Res, Oxy-Res, and Ac-Res by Hs68 and IMR-90 Cells

Cellular uptake was estimated by determining the remaining levels of Res and its analogs (20 *μ*M) during the incubation of these compounds with the cells for 0–7 days. In Table 6, the cellular uptake (i.e., 100% − the remaining level in %) of Res was quite low. For example, the remaining levels of Res in Hs68 and IMR-90 cells were 3.1% and 1.4%, respectively, on day 1 (*P* > 0.05 compared with the values on day 0). As the incubation time was prolonged, the uptake rates of Hs69 and IMR-90 cells on day 7 increased by approximately 6.8% and 9.2% (*P* < 0.05), respectively. The uptake rate of oxy-Res was larger than that of Res. Among the Res forms, ac-Res had the largest uptake rate; that is, its uptake rates were approximately 12.4% and 9.4% in Hs68 and IMR-90 cells, respectively on day 1 (*P* < 0.05). The uptake rate of ac-Res on days 3–7 corresponded to the uptake rate of Res because ac-Res was completely hydrolyzed to Res within 1 day.

### 3.12. Role of Res–FBS Interaction Mechanism and the Effect of Pyruvate

**Figure 8:**
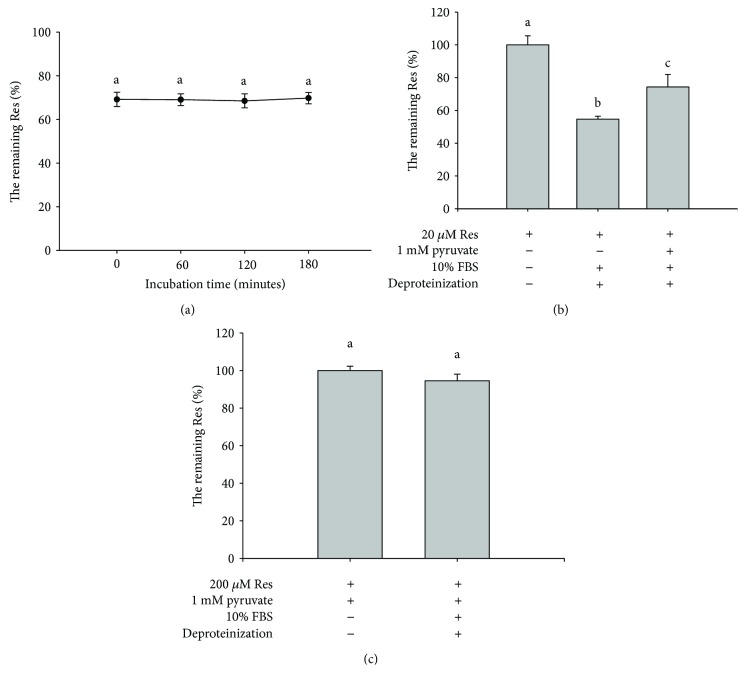
Effects of protein precipitation on the remaining levels of Res, oxy-Res, and ac-Res in the medium. (a) To evaluate the optimal time for the determination of the content of Res–FBS complex, we mixed 20 *μ*M Res with 10 mL of DMEM containing 10% FBS and 1 mM pyruvate. After incubation was performed at different time points, 100 *μ*L of the medium was treated with methanol to precipitate protein as in the procedures described in Materials and Methods. The remaining Res levels (%) were analyzed through HPLC. (b) To determine the effect of pyruvate on the content of Res–FBS complex, we mixed 20 *μ*M Res with 1 mL of DMEM with or without 10% FBS and 1 mM pyruvate. After deproteinization occurred, the remaining Res levels (%) were evaluated through HPLC. (c) We mixed 200 *μ*M Res with 1 mL of DMEM containing 1 mM pyruvate with or without 10% FBS. After deproteinization was completed, the remaining Res levels (%) were examined through HPLC. Values (mean ± standard deviation, *n* = 3) without a common letter are significantly different (*P* < 0.05).

The role of the Res–FBS interaction mechanism on the inhibition of Res degradation in DMEM was further investigated. The mechanism indicated that Res formed a complex with FBS and thus increased the stability of Res by preventing its degradation. Thus, we hypothesized that using protein precipitation with methanol would remove the Res–FBS complex and decrease the remaining level of Res in the deproteined medium. Thus, the remaining levels of Res in the deproteined medium should be utilized to evaluate the function of the Res–FBS interaction mechanism. In Figure 8(a), the remaining Res levels reached the maximum at 0 min and maintained at this value to 180 minutes (*P* > 0.05). The result showed that a brief time was enough for Res–FBS complex formation. In addition, pyruvate could decrease the Res–FBS interaction because the remaining Res levels increased compared with those in which 1 mM pyruvate was not added (Figure 8(b)). Approximately 45.4% ± 1.8% (i.e., bar 2; 100% − 54.6%) of Res could form a complex with FBS when 20 *μ*M Res was incubated in the medium containing 10% FBS but without 1 mM pyruvate (Figure 8(b)). Moreover, approximately 5.5% ± 3.6% (*P* > 0.05) of Res interacted with Res when 200 *μ*M Res was incubated with 10% FBS in DMEM (Figure 8(c)), suggesting that the role of Res–FBS interaction mechanism in the inhibition of Res degradation was limited at such a high concentration (i.e., 200 *μ*M Res). These results implied that a low Res concentration in the medium corresponded to a large role of the Res–FBS interaction mechanism for FBS to inhibit Res degradation.

## 4. Discussion

This study aimed to develop a method to inhibit Res degradation in medium under regular culture conditions and reveal the effects of Res on the lifespan of human Hs68 cells. In addition, we used two Res analogs, that is, oxy-Res and ac-Res, and an alternative fibroblast IMR-90 cell line to confirm the conclusions. The addition of ≥5 U/mL SOD into the medium can completely inhibit Res degradation in DMEM. The SOD activity of FBS was approximately 29.3 U/mL. Thus, the SOD activity of 10% FBS and the Res–FBS interaction mechanism in DMEM were insufficient to inhibit Res degradation completely. Moreover, catalase and pyruvate can potentiate SOD to scavenge superoxide. Consequently, the 10% FBS combined with pyruvate (1 mM) can completely inhibit the degradation of both Res and oxy-Res in DMEM under regular culture conditions. Ac-Res was completely hydrolyzed to Res within 24 h in DMEM under the same culture conditions. These results suggested that investigating the effects of ac-Res on cell lifespan was similar with investigating the effects of Res, although the extent of the effects was not the same because ac-Res exhibited higher uptake rate by both Hs68 and IMR-90 cells than Res. In contrast to our hypothesis, we observed that Res, oxy-Res, and ac-Res shortened the lifespan and induced the senescence of Hs68 cells in a concentration-dependent manner. Similar results were obtained with IMR-90 cells in MEM. To the best of our knowledge, this paper is the first to report the SOD activity of FBS and that pyruvate can potentiate the SOD activity and that 10% FBS combined with pyruvate (1 mM) under the regular culture conditions can completely inhibit the degradation of Res and oxy-Res in medium. However, we found that Res and its analogs shortened the lifespan of both Hs68 and IMR-90 cells.

Long et al. [34] reported that Res (approximately 1 mM) incubated in DMEM for 24 h generates no H_2_O_2_, which suggested that Res is not oxidized in DMEM. They also reported an inconsistent result that substantial amount of Res is degraded after 24 h of incubation [34]. The present study demonstrated that Res incubation in DMEM for 7 days degraded about 60% of Res, which confirmed that Res can be degraded by oxidation in DMEM. Res was also degraded in MEM. Given that cellular senescence is one of the most commonly used models for aging studies, a method to prevent the oxidative degradation of polyphenols in culture medium should be developed. SOD is an enzyme that can remove superoxide radicals catalytically by a dismutase reaction. Several studies proposed that polyphenol oxidation in vitro involves the generation of superoxide [35, 36]. In the present study, we observed that 10% FBS combined with pyruvate (1 mM) can completely avoid the degradation of Res in DMEM and MEM. Pyruvate is a compound often used in cell culture to improve cell growth. This compound can also eliminate H_2_O_2_ to avoid the artificial effects of H_2_O_2_ generated from Res oxidation. Thus, we used 10% FBS combined with pyruvate (1 mM) without the addition of SOD into the medium to avoid Res degradation and investigate the effects of Res and its analogs on the lifespan of Hs68 cells.

We also determined why pyruvate can potentiate SOD to scavenge superoxide. Using CuZnSOD as an example, SOD-catalyzed dismutation of superoxide can be written as the following reactions:

1. Cu^2+^ − SOD + O^2−^ → Cu^+^ − SOD + O_2_ (oxidation of superoxide)
2. Cu^+^ − SOD + O^2−^ + 2H^+^ → Cu^2+^ − SOD + H_2_O_2_ (reduction of superoxide)

According to the Le Chatelier's principle, which states that “whenever a system in equilibrium is disturbed, the system will adjust itself in such a way that the effect of the change will be nullified,” the equilibrium will direct to the right when H_2_O_2_ is scavenged by pyruvate or catalase. Consequently, a large amount of superoxide will be scavenged by SOD. Moreover, alkaline conditions are a major factor affecting polyphenol degradation in solution [19, 37]. Notably, the pH maintained by the sodium bicarbonate and the CO_2_ supply also played a crucial role in inhibiting Res degradation in the medium. Without CO_2_ supply, the pH of the culture media will increase and the degradation of Res and its analogs will also remarkably increase (data not shown). Although 10% CO_2_ is the recommended industrial standard for both DMEM and MEM, we regularly used 5% CO_2_ to culture Hs68 cells. The medium pH equilibrated with 5% CO_2_ must be higher than that equilibrated with 10% CO_2_. Our results showed that Res was stable in 5% CO_2_, which indicated that Res should also be stable in 10% CO_2_.

We postulated various mechanisms underlying the inconsistent effects of Res on the senescence or replicative lifespan of human cells. In addition to the difference in cell types used, another hypothesis is related to the stability of Res in medium. Res may cause senescence or lifespan shortening by the oxidation of Res in medium. Res oxidation will generate H_2_O_2_, which plays a vital role in causing cellular senescence. In this study, we found that Res was not degraded by oxidation in DMEM and MEM with 10% FBS and pyruvate (1 mM) under regular cultured conditions. Under such conditions, Res still induced the senescence and lifespan shortening of both Hs68 and IMR-90 cells. These results suggested that the senescence and lifespan-shortening effects of Res were the consequence of Res itself, not by the H_2_O_2_ generated through Res oxidation in media. Another possibility explaining the inconsistent effects of Res on cellular senescence is the biphasic hormetic dose response at the cellular level. Many of the Res-induced effects are dependent on dose, and opposite effects occur at low and high doses, a phenomenon called hormesis [38]. If we select a Res concentration that is considerably high, then the Res benefit may not be evident and it may exhibit an opposite effect. Nonetheless, we observed that Res exerted no hormetic effect on the lifespan of Hs68 and IMR-90 cells at the Res concentrations ranging within 0.5–20 *μ*M. Therefore, our results demonstrated that Res cannot extend the lifespan of Hs68 and IMR-90 cells. By contrast, Res can induce the lifespan shortening of Hs68 and IMR-90 cells. Hence, these results displayed no support to the hypothesis that Res possesses CRM potential at the cellular level. Indeed, reports about the effects of Res on the lifespan of organisms are also inconsistent. Some studies demonstrated that Res extends the lifespan of yeast, *C. elegans*, *Drosophila*, and the short-lived fish *Nothobranchius furzeri* [39, 40]. Other studies indicated that Res cannot extend the lifespan of *Drosophila* [41] or mice [42] and it exerts variable effects on the lifespan of *C. elegans* [41].

Res demonstrates multiple functionalities. Similarly, oxy-Res exhibits many health-promoting properties, such as anti-inflammation, antioxidation, antiobesity, cholesterol-lowering, antivirus, and neuroprotective effects [23]. Ac-Res possesses lower antioxidative activity but higher bioavailability than Res [27]. In the present study, we found no evidence of the lifespan-extending effects of these molecules on Hs68 cells. However, we observed a biphasic hormetic dose response of Res, oxy-Res, and ac-Res on their cyototoxicity in Hs68 and IMR-90 cells. These results suggested that Res and its analogs at a low concentration may still exhibit some beneficial effects induced by a mechanism similar to the hormetic effect. Although we did not address the mechanism related to the hormetic effect caused by Res, a recent report showed that the hormetic effect of Res on cell viability is due to the prooxidative action of Res to trigger the genomic and metabolic shifts, thereby causing the hormetic shifting of cellular defense toward a considerably reductive state to improve the physiological resilience to oxidative stress [43]. Alam et al. [32] also demonstrated that Res displays high absorption (>70%) but low bioavailability after oral administration in humans, which indicated a rapid and extensive metabolism of oral Res. Res can be dissolved in blood at concentrations of several hundreds of *μ*M (the solubility of Res in water is less than 0.05 mg/mL or 220 *μ*M), but its physiological level in blood is less than 3 *μ*M. These findings suggested that most Res absorbed from the gastrointestinal tract will be metabolized in the body. The authors believed that the rapid and extensive metabolism of Res may contribute to the detoxification of Res in vivo. The metabolites may also contribute to the multiple functionalities of Res. Further investigations should focus on the CRM potential of Res metabolites in vivo at a cellular level.

Considering that Res is a component with health benefits, we were not interested in revealing its mechanism of inducing the senescence of Hs68 cells, investigating the activity and expression levels of the principal enzymes, such as SOD, catalase, and glutathione peroxidase, involved in oxidative stress response, or detecting the underlying signaling molecules. We were interested in determining the role of the Res–FBS interaction mechanism of the inhibition of Res degradation in DMEM by FBS. Our results suggested that the role of Res–FBS interaction mechanism in the inhibition of Res degradation was minimal when the concentration of Res incubated in 10% FBS-containing medium was high. In addition, the Res–FBS complex involves noncovalent interactions, such as hydrogen bonding, van der Waals forces, hydrophobic interactions, and electrostatic interactions [21]. We found that pyruvate could decrease the Res–FBS complex in the medium, suggesting that pyruvate might increase the partition of Res in the medium by decreasing the polarity of the medium. Thus, the SOD activity in FBS to inhibit Res degradation became important when 1 mM pyruvate was added to the medium. These results implied that the SOD activity was necessary to avoid the degradation of Res molecules that did not form a complex with FBS, such as free Res in the medium. The Res–FBS interaction mechanism also played a role in the stability of Res, especially when the Res concentration was low.

## 5. Conclusion

In conclusion, the SOD activity could completely prevent Res degradation in the medium. FBS could inhibit Res degradation in the medium through SOD activity and Res–FBS interaction. Pyruvate could potentiate the SOD activity of FBS. Therefore, 10% FBS combined with 1 mM pyruvate possessed a sufficient SOD activity to inhibit Res and oxy-Res degradation completely in the medium under regular culture conditions. Ac-Res was hydrolyzed to Res within 24 h in DMEM under the same conditions. Although a method to avoid Res degradation was developed, Res, oxy-Res, and ac-Res shortened the lifespan of human Hs68 and IMR-90 cells. These results did not support the hypothesis that Res showed a CRM potential at a cellular level.

## Figures and Tables

**Table 1:** Effect of SOD on Res degradation in DMEM with or without FBS.

	SOD activity (U/mL)			
	0	0.5	5	50
Without FBS				
Day 0	100 ± 2.6^a^	100 ± 1.4^a^	100 ± 5.7^a^	100 ± 5.5^a^
Day 1	76.0 ± 2.4^b^	94.6 ± 2.1^b^	97.6 ± 2.2^a^	101.9 ± 1.6^a^
Day 7	37.9 ± 1.5^c^	67.4 ± 4.7^c^	96.7 ± 0.9^a^	99.5 ± 4.3^a^
With FBS				
Day 0	100 ± 2.0^a^	100 ± 4.3^a^	100 ± 2.6^a^	100.0 ± 5.8^a^
Day 1	95.6 ± 1.2^b^	97.8 ± 3.1^a^	101.3 ± 4.2^a^	98.5 ± 1.8^a^
Day 7	79.4 ± 1.4^c^	89.6 ± 2.0^b^	98.4 ± 3.7^a^	97.1 ± 2.8^a^

Res at 200 *μ*M was incubated for 0–7 days in DMEM, which contained 3.7 g/L sodium bicarbonate with different doses of SOD with or without 10% FBS at 37°C in a CO_2_ incubator supplied with 5% CO_2_. The remaining Res (%) levels related to the peak area of Res on day 0 were determined by HPLC. Values (mean ± standard deviation; *n* = 3) in the same column without a common letter are significantly different (*P* < 0.05).

**Table 2:** Effect of catalase or pyruvate on Res degradation in DMEM.

	Catalase	Pyruvate
Day 0	100 ± 2.5^a^	100 ± 4.7^a^
Day 1	84.8 ± 3.7^b^	56.4 ± 2.9^b^
Day 7	28.6 ± 3.4^c^	10.4 ± 3.1^c^

Res at 200 *μ*M was incubated for 0–7 days in DMEM, which contained 3.7 g/L sodium bicarbonate with 100 U/mL catalase or pyruvate (1 mM) at 37°C in a CO_2_ incubator supplied with 5% CO_2_. The remaining Res (%) levels related to the peak area of Res on day 0 were determined by HPLC. Values (mean ± standard deviation; *n* = 3) in the same column without a common letter are significantly different (*P* < 0.05).

**Table 3:** Additional SOD required to inhibit Res degradation in DMEM containing FBS and catalase or pyruvate.

	SOD activity (U/mL)			
	0	0.5	5	50
FBS + catalase				
Day 0	100.0 ± 1.5^a^	100 ± 2.3^a^	100 ± 5.1^a^	100 ± 2.6^a^
Day 1	102.4 ± 1.6^a^	98.4 ± 1.9^a^	102.4 ± 2.9^a^	97.6 ± 5.2^a^
Day 7	101.0 ± 1.1^a^	99.3 ± 2.7^a^	101.3 ± 4.7^a^	98.3 ± 2.1^a^
FBS + pyruvate				
Day 0	100 ± 1.0^a^	100 ± 1.8^a^	100 ± 3.6^a^	100 ± 2.9^a^
Day 1	97.3 ± 5.6^a^	101.4 ± 3.7^a^	98.6 ± 2.8^a^	101.2 ± 2.5^a^
Day 7	104 ± 1.9^a^	96.5 ± 4.3^a^	99.7 ± 3.4^a^	97.3 ± 4.6^a^

Res at 200 *μ*M was incubated for 0–7 days in DMEM, which contained 3.7 g/L sodium bicarbonate, 10% FBS, and 100 U/mL catalase (FBS + catalase) or 1 mM pyruvate (FBS + pyruvate) combined with different doses of SOD at 37°C in a CO_2_ incubator supplied with 5% CO_2_. The remaining Res levels (%) related to the peak area of Res on day 0 were determined by HPLC. Values (mean ± standard deviation; *n* = 3) in the same column without a common letter are significantly different (*P* < 0.05).

**Table 4:** Effect of FBS combined with pyruvate on Res degradation in MEM.

	Control	+FBS	+FBS +Pyruvate	+Pyruvate
Day 0	100 ± 0.2^a^	100 ± 2.0^a^	100 ± 6.6^a^	100 ± 2.2^a^
Day 1	89.0 ± 1.9^b^	94.5 ± 1.4^b^	97.6 ± 0.3^a^	78.3 ± 4.3^b^
Day 7	69.0 ± 1.8^c^	80.3 ± 2.7^c^	97.5 ± 0.5^a^	57.6 ± 1.4^c^

Res at 200 *μ*M was incubated for 0–7 days in minimum essential medium (MEM), which contained both or either 2.2 g/L sodium bicarbonate with 10% FBS (+FBS) and 1 mM pyruvate (+pyruvate) at 37°C in a CO_2_ incubator supplied with 5% CO_2_. The remaining Res levels (%) related to the peak area of Res on day 0 were determined by HPLC. Values (mean ± standard deviation; *n* = 3) in the same column without a common letter are significantly different (*P* < 0.05).

**Table 5:** Degradation of Res and oxy-Res in DMEM and MEM with FBS and pyruvate under regular culture conditions.

	Res (%)	Oxy-Res (%)
DMEM		
Day 0	100 ± 1.8^a^	100 ± 2.7^a^
Day 1	104.1 ± 2.3^a^	97.5 ± 3.0^a^
Day 7	111.5 ± 0.5^a^	97.3 ± 2.8^a^
MEM		
Day 0	100 ± 5.7^a^	100 ± 4.2^a^
Day 1	102.9 ± 2.3^a^	98.3 ± 5.1^a^
Day 7	102.8 ± 3.1^a^	94.5 ± 3.7^a^

Res and oxy-Res were incubated for 0–7 days in media, which contained 3.7 g/L sodium bicarbonate and antibiotics for DMEM, 2.2 g/L sodium bicarbonate, 0.1 mM nonessential amino acids, and antibiotics for MEM with 10% FBS and pyruvate (1 mM) at 37°C in a CO_2_ incubator supplied with 5% CO_2_. The remaining levels of Res and oxy-Res (%, related to the peak area on day 0) were determined by HPLC. Values (mean ± standard deviation; *n* = 3) in the same column without a common letter are significantly different (*P* < 0.05).

**Table 6:** Cellular uptake of Res, oxy-Res, and ac-Res for Hs68 and IMR-90 cells in the medium.

	Res (%)	Oxy-Res (%)	Ac-Res (%)
Hs68 cells			
Day 0	100.0 ± 4.7^a^	100.0 ± 3.6^a^	100.0 ± 5.2^a^
Day 1	96.9 ± 4.9^a^	99.4 ± 3.3^a^	^#^87.6 ± 0.4^b^
Day 3	96.2 ± 5.6^a^	89.3 ± 1.1^b^	^#^86.4 ± 2.4^b,c^
Day 7	93.2 ± 1.4^a^	75.3 ± 5.0^c^	^#^82.7 ± 2.3^c^
IMR-90 cells			
Day 0	100 ± 5.1^a^	100 ± 6.8^a^	100 ± 3.0^a^
Day 1	98.6 ± 4.0^a^	101.2 ± 1.0^a^	^#^90.6 ± 1.9^b^
Day 3	98.3 ± 3.9^a^	93.5 ± 3.2^a^	^#^90.2 ± 2.1^b^
Day 7	90.8 ± 0.9^b^	83.5 ± 3.9^b^	^#^84.4 ± 1.1^c^

Hs68 and IMR-90 cells (1 × 10^5^) were incubated with Res, oxy-Res, and ac-Res for 0–7 days in DMEM and MEM, respectively, with 10% FBS and 1 mM pyruvate under the regular culture conditions. The remaining levels of Res, oxy-Res, and ac-Res (%; related to the concentration on day 0) in the media were determined through HPLC. The standard curves of Res, oxy-Res, and ac-Res were established for quantifications. The remaining level is inversely proportional to the cellular uptake. Values (mean ± standard deviation; *n* = 3) in the same column without a common letter are significantly different (*P* < 0.05). ^#^ac-Res was completely hydrolyzed to Res after day 1. As such, the uptake of ac-Res on days 1–7 was revealed by quantifying the newly formed Res peak in the calibration curve of Res.
